# Author Correction: Structural analysis of human ARS2 as a platform for co-transcriptional RNA sorting

**DOI:** 10.1038/s41467-018-04643-5

**Published:** 2018-05-31

**Authors:** Wiebke Manuela Schulze, Frank Stein, Mandy Rettel, Max Nanao, Stephen Cusack

**Affiliations:** 1 European Molecular Biology Laboratory, Grenoble Outstation, 71 Avenue des Martyrs, CS 90181, Grenoble Cedex 9, 38042 France; 2 European Molecular Biology Laboratory, Heidelberg, Meyerhofstraße 1, Heidelberg, 69117 Germany; 3 Present Address: ESRF-The European Synchrotron, Avenue des Martyrs 71, CS40220, Grenoble Cedex 9, 38043 France

Correction to: *Nature Communications* 10.1038/s41467-018-04142-7, published online 27 April 2018

The previously published version of this Article contained an error in Fig. 1. In panel d, the Arabidopsis SERRATE protein was incorrectly labelled ‘Human SERRATE’ and should have been labelled ‘SERRATE’. The error has been corrected in both the PDF and HTML versions of the Article.

