# Supplementary material for: Structural analysis of human ARS2 as a platform for co-transcriptional RNA sorting
Source: Nat Commun. 2018 Apr 27;9:1701. doi: 10.1038/s41467-018-04142-7 (PMC5923425; doi:10.1038/s41467-018-04142-7)
Supplement: Supplementary file 1 — Supplementary Information [file 41467_2018_4142_MOESM1_ESM.pdf]

Structural analysis of human ARS2

a platform for co-transcriptional RNA sorting

Schulze *et al*.

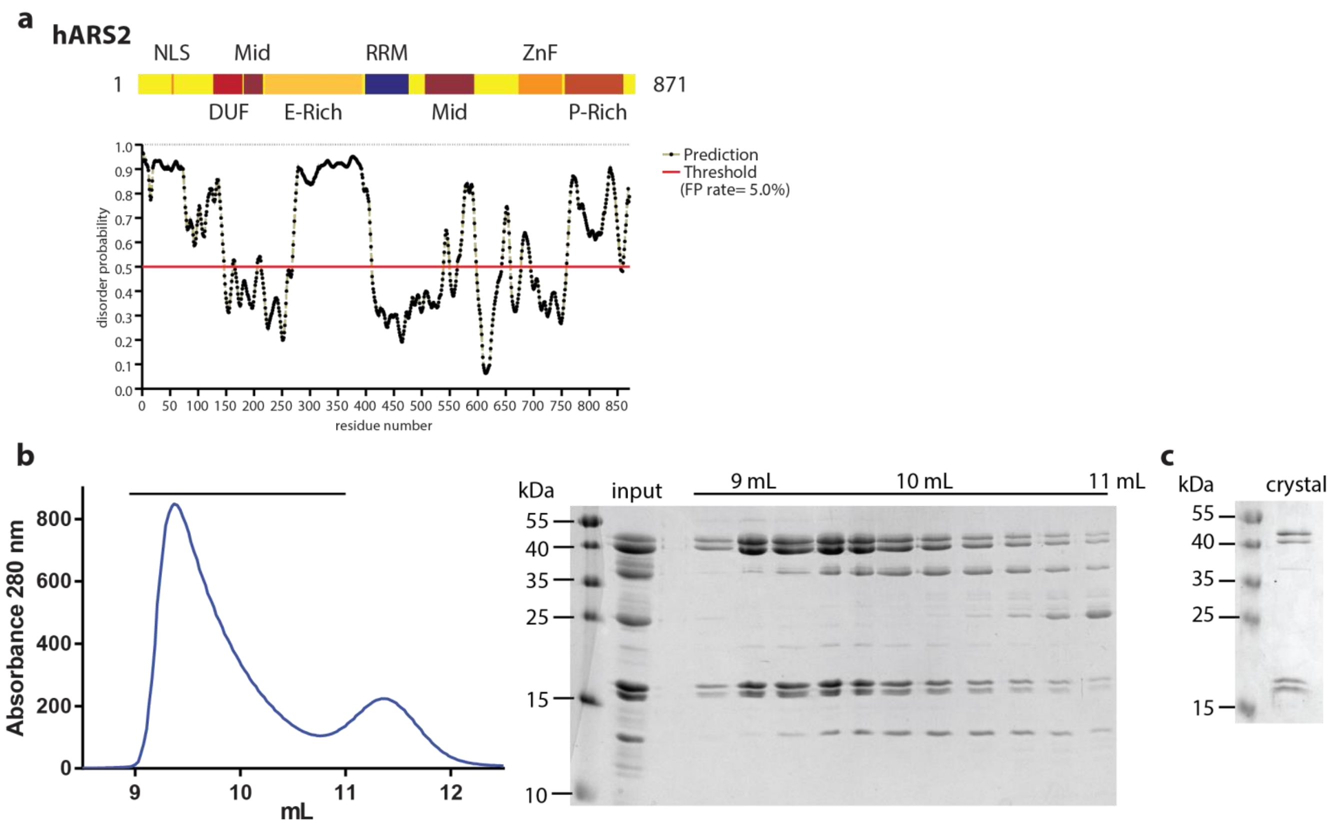

Supplementary Figure 1: The core of human ARS2 is composed of two co-folding structured regions.

a: Schematic diagram of the domain structure of hARS2[1] (top) and disorder prediction of hARS2 performed with PrDos[2] (bottom). NLS: nuclear localisation sequence; DUF: domain of unknown function; RRM: RNA recognition motif; ZnF: zinc finger; FP rate: false positive rate of prediction.

b: Chromatogram (left) and Coomassie-stained SDS-PAGE (right) of trypsinated hARS2. $ARS2^{147\text{-}763}$ was digested with trypsin and then subjected to gel filtration. LC-ESI TOF analysis of co-eluting doublets resulted in the identification of fragments that were assigned to residues 147-285, 147-286, 147-290 (bottom doublet) and 393-763 and 405-763 (top doublet). This shows that the double bands result from cleavage at either residue 285/6 or 290 and 393 or 405. Based on this result as well as the disorder prediction (a), the residues 147-270 and 408-763 were used for co-expression and further experiments.

c: Silver-stained SDS-PAGE of a crystal obtained from the purified peak fraction of trypsinated $ARS2^{147\text{-}763}$ shown in (b).

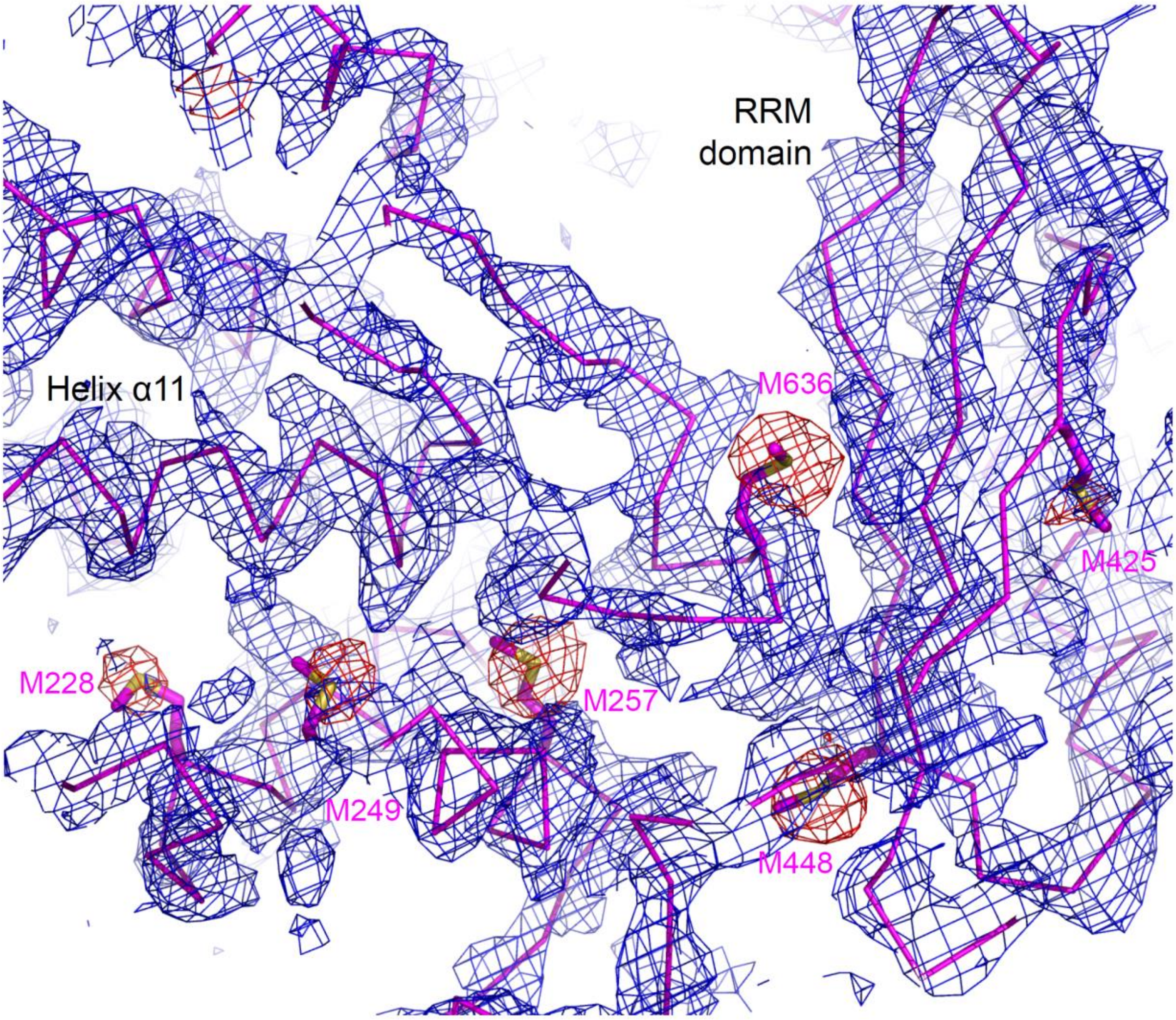

Supplementary Figure 2: Experimental map of ARS2$^{171\text{-}270+408\text{-}763}$ in P$6_5$22 space-group.

SIRAS phased map contoured at 1.2 σ (blue), after density modification with PARROT. Anomalous difference map contoured at 4.5 σ (red), phased with refined model (magenta backbone and sidechains) showing density for selenomethionine positions.

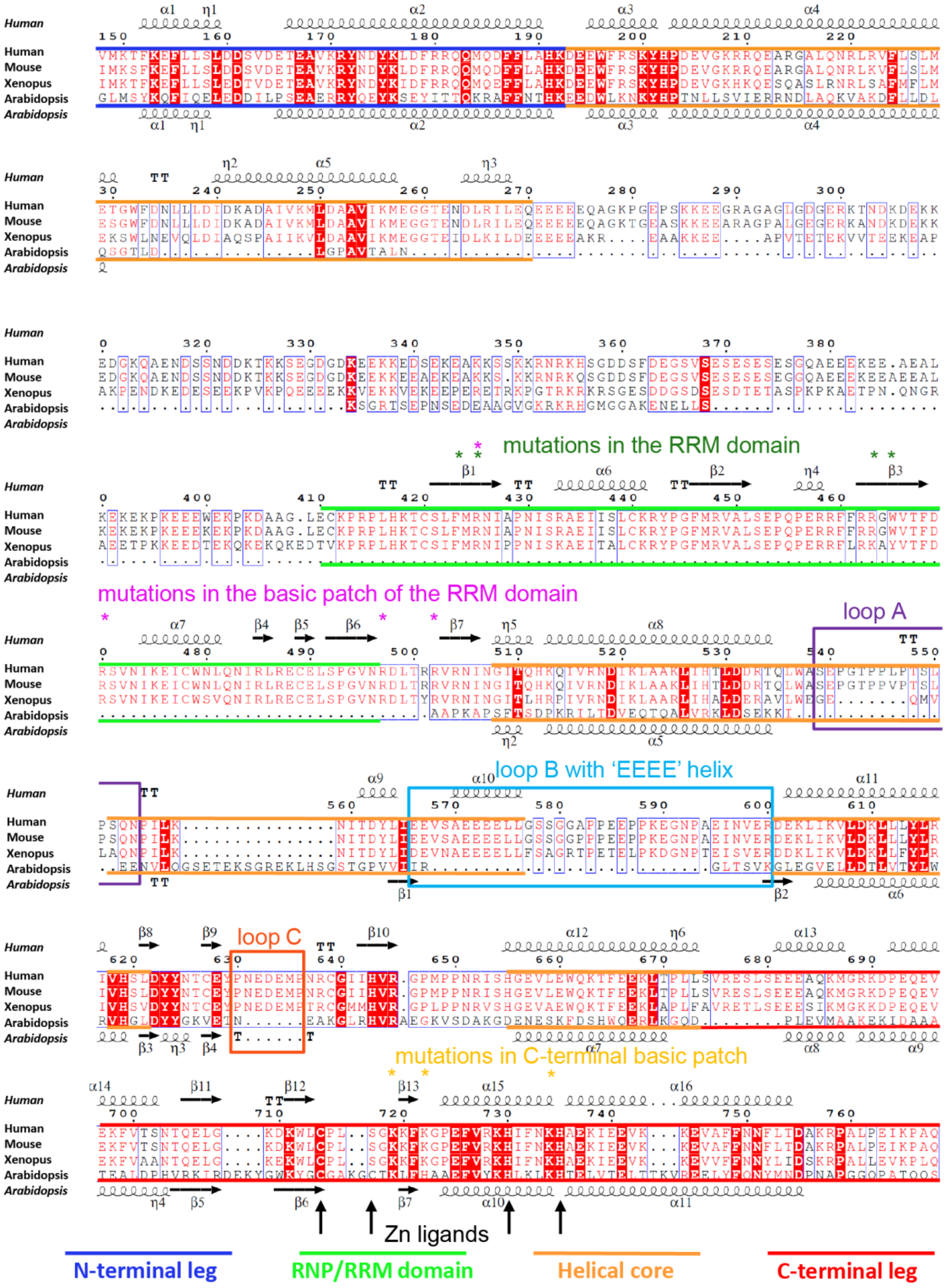

Supplementary Figure 3: Structure-based alignment of ARS2 protein sequences. Structure-based sequence alignment drawn with ESPRIPT[3] of human, mouse, *Xenopus* ARS2 and *Arabidopsis thaliana* SERRATE with the corresponding crystallographically determined

secondary structures above (hARS2, this work) and below (SERRATE, PDB: 3AX1[4]). The different domains of hARS2 are highlighted in colour (see also Figure 1a, b) together with the metazoan specific loop A (538-552), loop B (568-598) containing the ‘EEEE helix’ and loop C (630-637). Note that the RRM domain is only conserved in metazoans and *S. pombe*. Arrows indicate residues in the C-terminal arm involved in zinc binding in *A. thaliana*. Positions of residues mutated for functional studies are marked by an asterisk above the alignment: two quadruple mutants in the RRM domain (dark green and magenta) and the triple lysine mutant in the C-terminal basic patch (yellow).

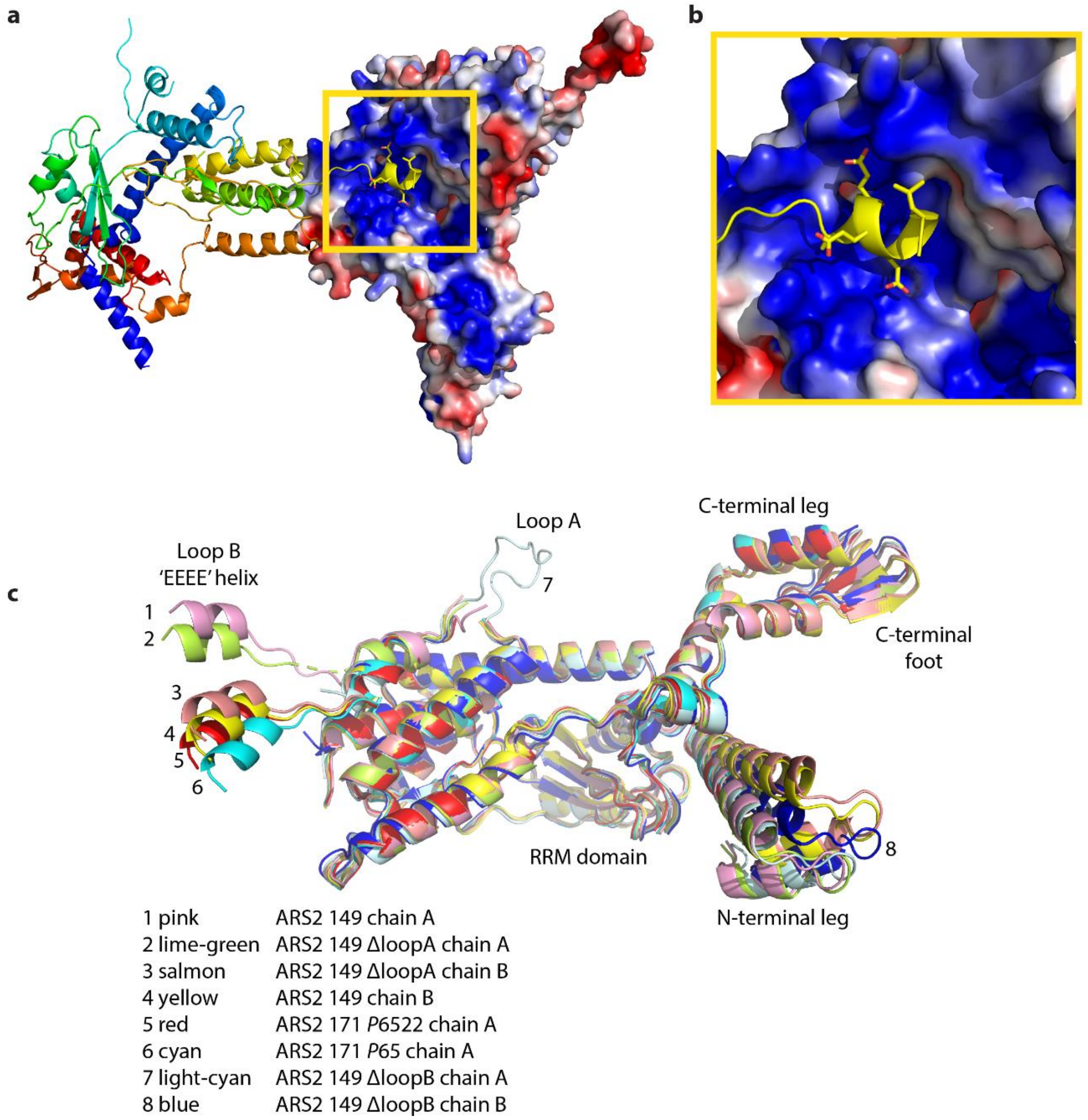

Supplementary Figure 4: Crystal contacts and flexibility of hARS2.

a: Crystal contact between by acidic `EEEE' helix of loop B binding onto the basic RRM β-sheet surface of a neighbouring molecule.

b: Close-up view of the crystal contact made by acidic `EEEE' helix binding onto the basic RRM β-sheet surface of a neighbouring molecule (a).

c: Superposition of eight different human ARS2 structures determined in different crystal forms as indicated showing adaption of the orientation of the loop bearing the `EEEE' helix in order to preserve the crystal contact shown in (a) and (b). Note that in the structure where loop B was deleted, loop A is observed in chain A (structure 7).

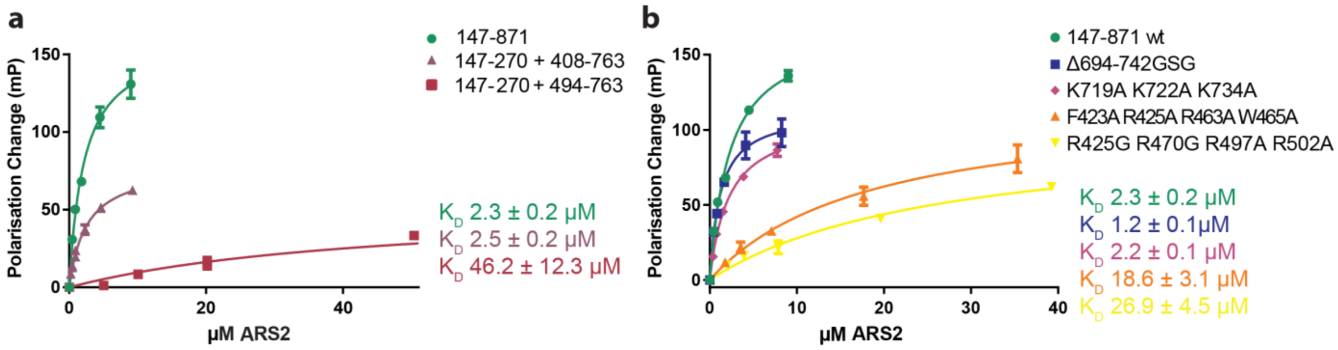

Supplementary Figure 5: The RRM domain of hARS2 binds ssRNA.

FAM-labelled RNA was used to determine the RNA binding of ARS2 by fluorescence polarisation. Curves show all data points measured. The K_D was derived by fitting the data to a single site binding model. Error bars show SD of three experiments.

a: $ARS2^{147-871}$, $ARS2^{147-270+408-763}$ and $ARS2^{147-270+494-763}$ were titrated to FAM-labelled ssRNA and the fluorescence polarisation was measured.

b: Deletion and site specific mutants of $ARS2^{147-871}$ were titrated to FAM-labelled ssRNA to more precisely localise the RNA binding site.

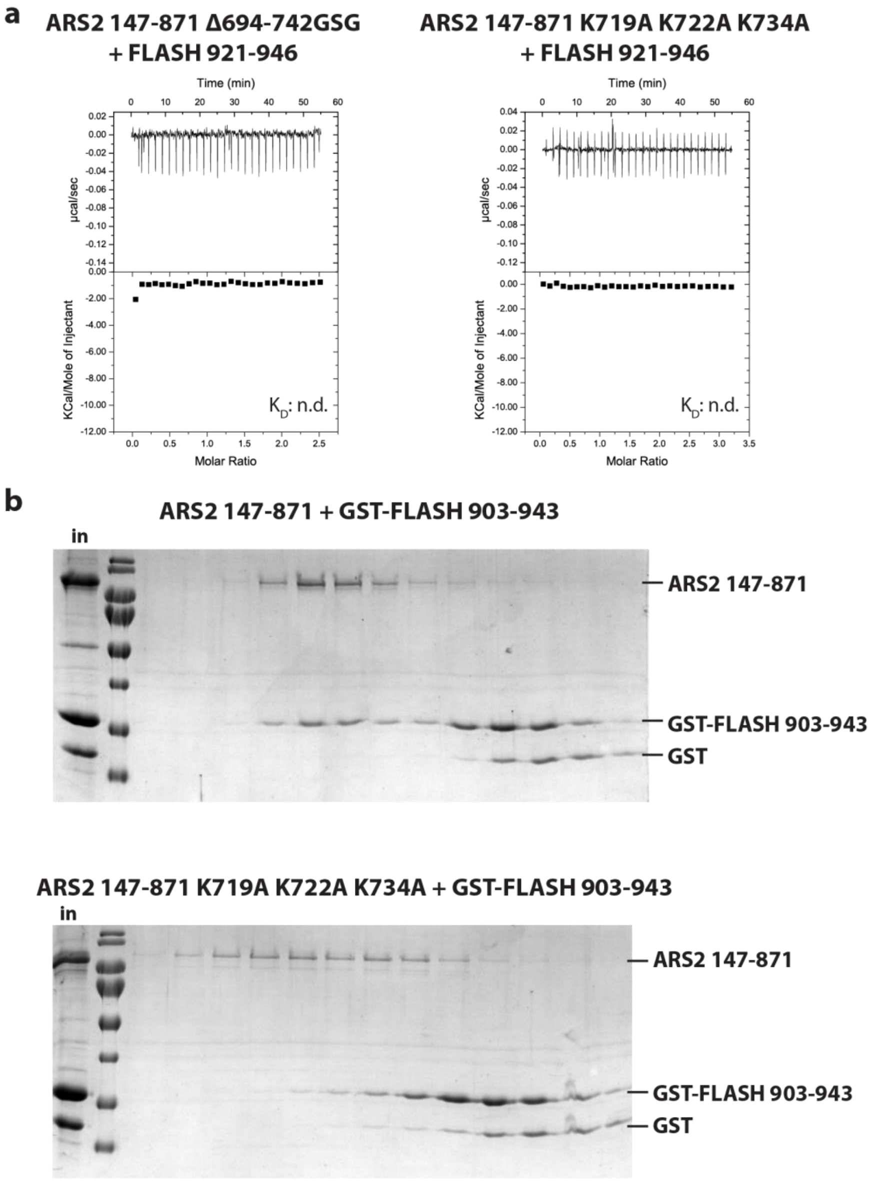

Supplementary Figure 6: The C-terminal leg of ARS2 is required for interaction with FLASH.

a: Isothermal titration calorimetry data of FLASH 921-946 binding to ARS2. FLASH$^{921\text{-}946}$ in the syringe was titrated to ARS2$^{147\text{-}871}$ constructs in the cell. Binding is not observed for the C-terminal leg deletion (Δ694-742) or the triple lysine mutant (K719A K722A K734). The upper graphs show the raw data and the bottom graphs show the ligand concentration dependence of the heat released upon binding after normalization. K_D values represent the average from at least two independent experiments (see Table 2).

b: Coomassie-stained SDS-PAGE of SEC of ARS2$^{147\text{-}871}$ wild type (upper) and ARS2$^{147\text{-}871\ K719A\ K722A\ K734}$ (lower) with GST-FLASH$^{903\text{-}943}$. Recombinant purified proteins were mixed, subjected to SEC and protein-containing fractions were analysed by Coomassie-stained SDS-PAGE. FLASH only binds to the wild type ARS2.

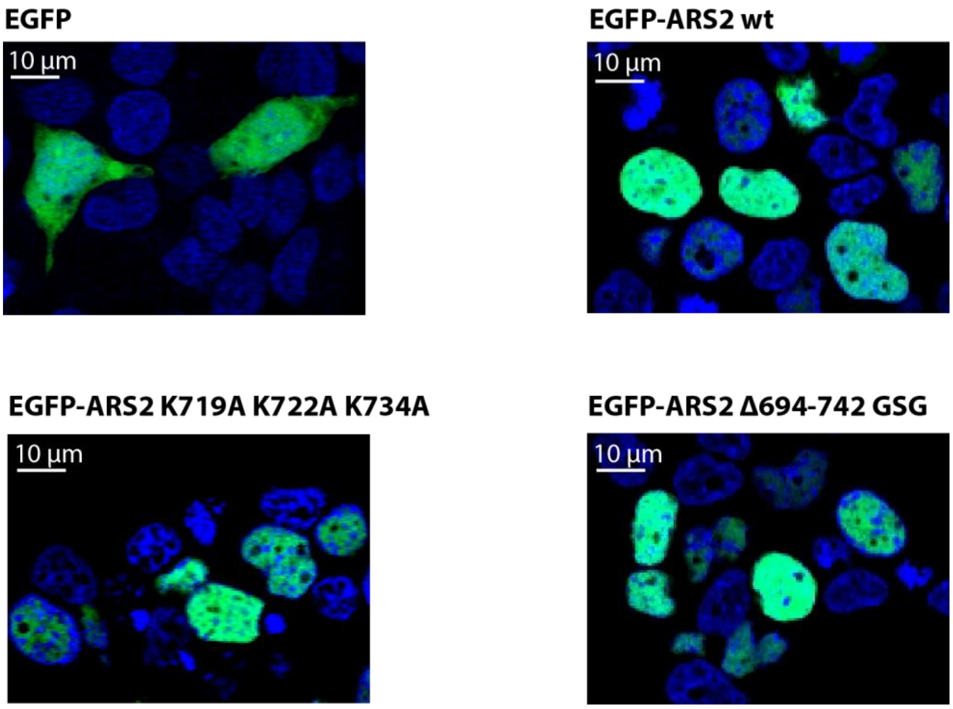

Supplementary Figure 7: Subcellular localisation of EGFP-ARS2 constructs within HEK 293/T17 cells.

30 h post transfection with EGFP or EGFP-ARS2 constructs, HEK 293/T17 cells were fixed, permeabilised and DAPI stained prior to fluorescence microscopy. EGFP: green, DAPI: blue.

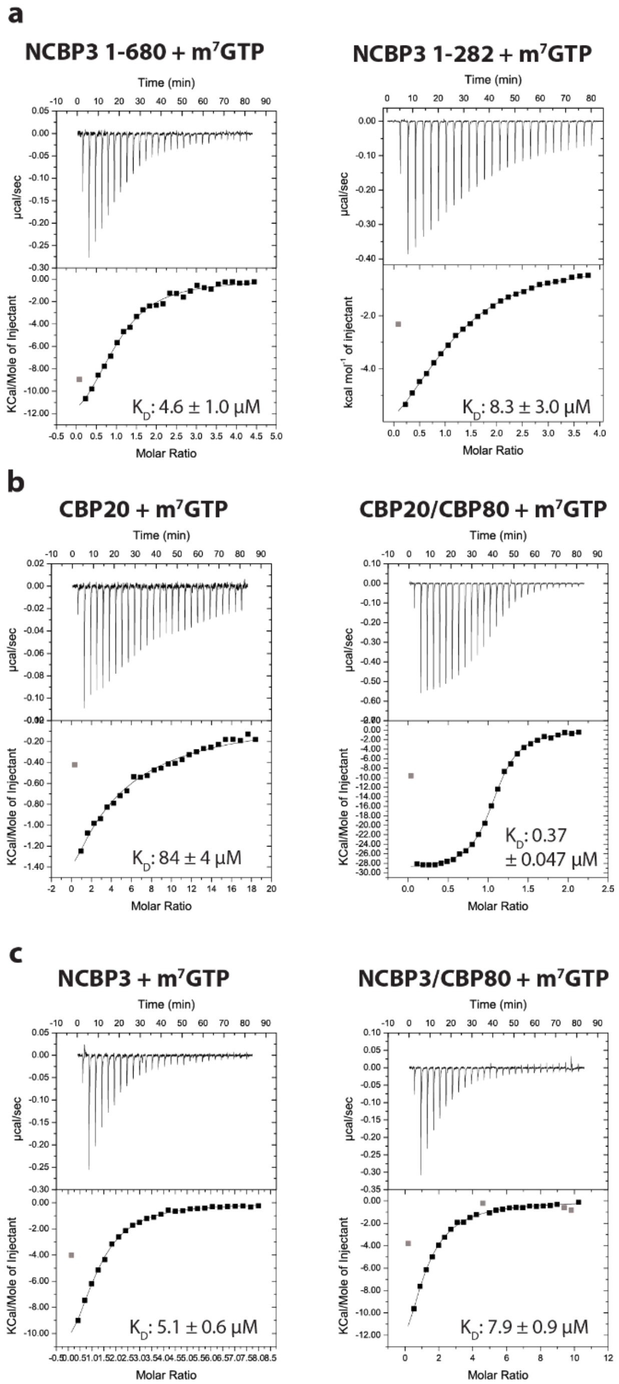

Supplementary Figure 8: ITC data of m^7GTP binding to NCBP3 or CBP20 and the effect of added CBP80.

a: m^7GTP in the syringe was titrated to full-length NCBP3 or NCBP3$^{1\text{-}282}$ in 120 mM NaCl.

b: m^7GTP was titrated to CBP20 or (c) NCBP3 in the presence or absence of 2 fold molar excess of CBP80 at 250 mM NaCl.

The upper graphs show the raw data and the bottom graphs show the ligand concentration dependence of the heat released upon binding after normalization. K_D values represent the average from at least two independent experiments (see Table 2).

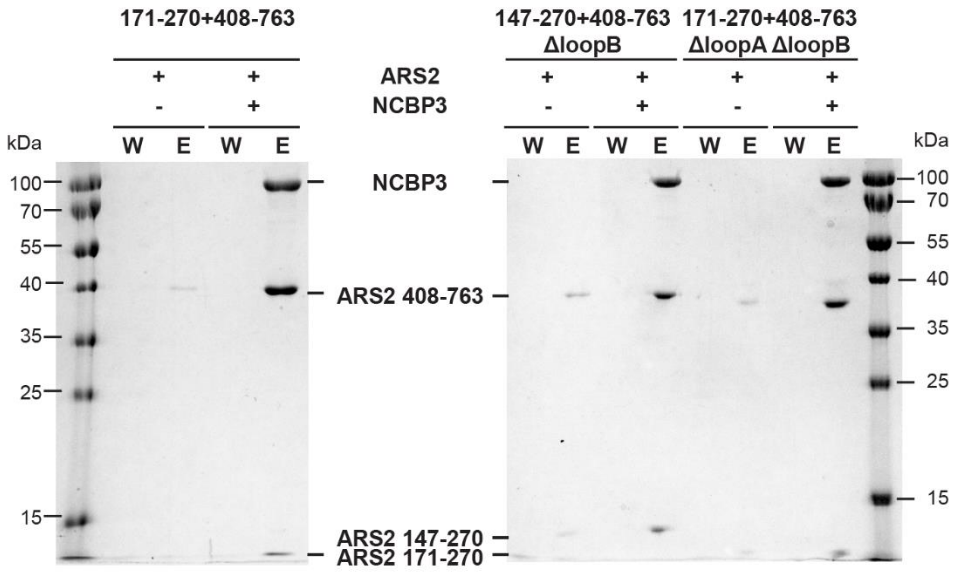

Supplementary Figure 9: NCBP3 binding to truncated ARS2 constructs. m^{7}GTP pull down of full length NCBP3 with different ARS2 constructs used for crystallisation as indicated (ARS2$^{171\text{-}270+408\text{-}763}$, ARS2$^{147\text{-}270+408\text{-}763\Delta loopB}$, ARS2$^{171\text{-}270+408\text{-}763\Delta loopA\Delta loopB}$). Purified recombinant NCBP3 was immobilised on m^{7}GTP sepharose before incubation with ARS2. After extensive washing the last wash (W) and eluted (E) fractions were analysed by Commassie-stained SDS-PAGE.

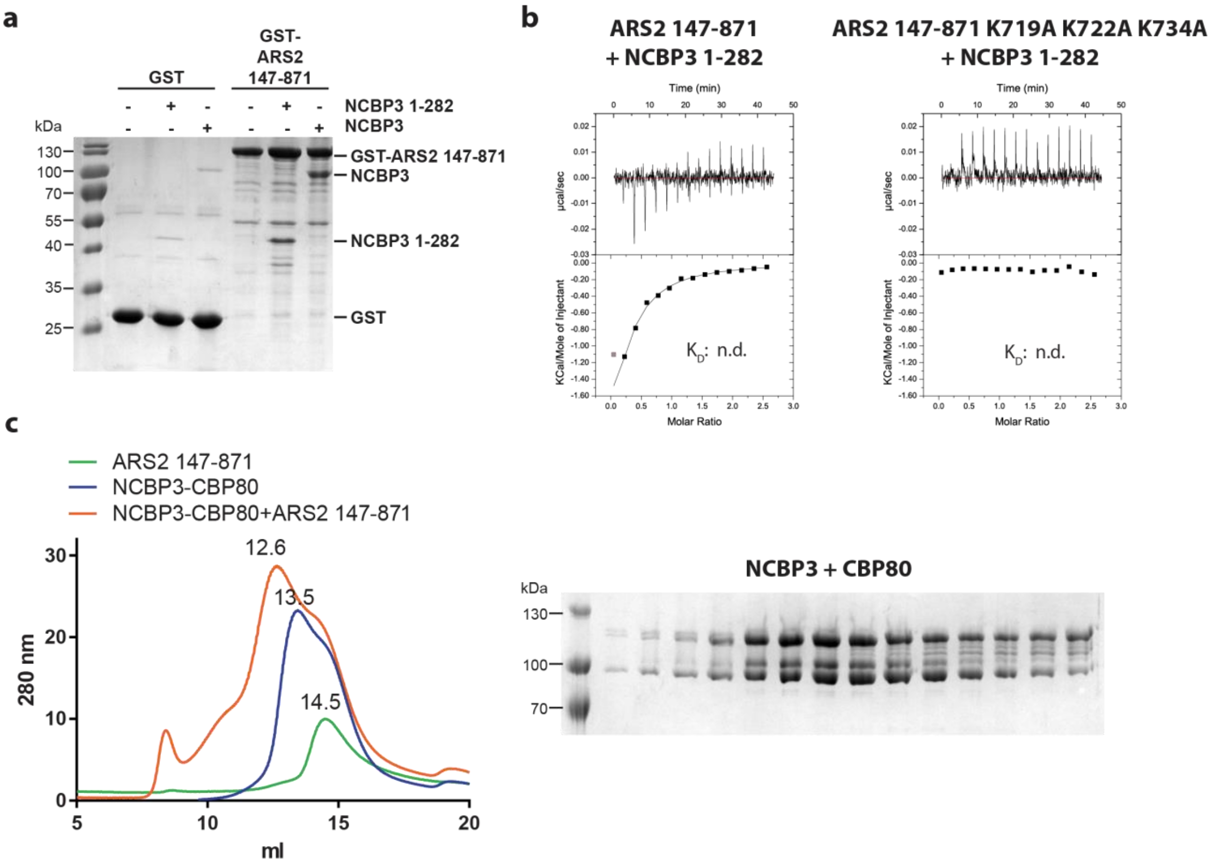

Supplementary Figure 10: NCBP3 binding studies with ARS2 and CBP80.

a: GST pull down of GST-ARS2 and NCBP3. GST-ARS2$^{147\text{-}871}$ or GST as control was immobilised on glutathione resin followed by incubation with full-length NCBP3 or NCBP3$^{1\text{-}282}$. After extensive washing the eluted fractions were analysed by Commassie-stained SDS-PAGE.

b: Isothermal titration calorimetry data for NCBP3 binding to ARS2. NCBP3$^{1\text{-}282}$ in the syringe was titrated to ARS2$^{147\text{-}871}$ or ARS2$^{147\text{-}871\ K719A\ K722A\ K734A}$ in the cell. The ITC experiments were carried out in buffer containing 250 mM NaCl and with 2.5 µl injections. The upper graphs show the raw data and the bottom graphs show the ligand concentration dependence of the heat released upon binding after normalisation. Note that although no K_D could be obtained, binding of NCBP3 to ARS2 wild type was observed, as indicated by the measured heat release, but not to the triple lysine mutant.

c: Size exclusion chromatogram and Coomassie-stained SDS-PAGE of NCBP3-CBP80-ARS2. Purified recombinant ARS2$^{147\text{-}871}$, NCBP3-CBP80 or NCBP3-CBP80+ARS2$^{147\text{-}871}$ were subjected to gel filtration. Their elution profiles were overlaid (left) and individual fractions of NCBP3-CBP80+ARS2 were analysed by SDS-PAGE (right).

Supplementary Table 1: Primers used in this work.

**Primer**	**Sequence 5' to 3'**
**ARS2**	
ARS2 147 fw (NcoI)	CATGCCATGGGCCCCGTGATGAAGACCTTCAAGGAGT
ARS2 171 fw (NcoI)	CTTTATTTTCAGGGCGCCATGCGCTATAATGACTACAAGC TGGATTTC
ARS2 408 fw (NcoI)	CATGCCATGGGGCTGGAGTGCAAGCCGC
ARS2 494 fw (NcoI)	CATGCCATG GGTGTGAACAGGGACCTGACC
ARS2 270 rev (XhoI)	GAATCTCGAGTCACTGCTCCAGGATGCGAAGATCATTC
ARS2 763 rev (XhoI)	GAATCTCGAGTCACTCAGGCAGAGCTGGGCG
ARS2 rev (XhoI)	GAATCTCGAGTCAAAAGAAATCAACATCGT
**FLASH**	
pETM30 FLASH 903-943 fw	CCTATCTATAAGAGCGATAAATGCACTGAAGCAGATACA TGCAAAAACTCACCCTTAGATGAACTGGAAGAAGGCGA ATTC
pETM30 FLASH 903-943 rev	TTTATC GCTCTTATAGATAGGCTTCTGATTCTCTTTATTCAGATCG GACATGGCGCCCTGAAAATAAAGATTC
**EGFP constructs pcDNA3.1**	
EGFP fw (HindI)	CACTATAGGGAGACCCAAGCTATGGTGAGCAAGGGCGA GG
EGFP GSGGGS rev (XbaI)	CTATAGAATAGGGCCCTCTAGTCA GGAGCCGCCTCCGCTGCC CTTGTACAGCTCGTCCATGC
ARS2 rev (XbaI)	CTATAGAATAGGGCCCTCTAGTCAAAAGAAATCAACATC GTCTGGGGC
EGFP GSGGGS ARS2 fw	CTGTACAAGGGCAGCGGAGGCGGCTCCATGGGTGACAGT GATGACGAGTAC
EGFP GSGGGS rev	GGAGCCGCCTCCGCTGCCCTTGTACAGCTCGTCCATGC
**NCBP3**	
NCBP3 fw pFAST (NcoI)	CTGTATTTTCAGGGCGCCATGGCGGCCGTACGGGGC
NCBP3 fw pETM11	CTTTATTTTCAGGGCGCCATGGCGGCCGTACGGGGC

(NcoI)	
NCBP3 282 rev (XhoI)	GGTGGTGGTGGTGCTCGAGTCATTTCATGTAATACTGACTTCTTCTGG
NCBP3 rev (XhoI)	TTGGTACCGCATGCCTCGAGTCAGGACTCTGCCTCTGAACC
**Mutagenesis**	
**ARS2**	
K719A K722A fw	GCCAAATTCGCCGGTCCT GAGTTTGTGCGCAAACATATC
K719A K722A rev	AGGACCGGCGAATTTGGCGCCACTGAGAGGACAC
K734A fw	CTTCAACGCCCATGCAGAGAAAATTGAGGAAG
K734A rev	GCATGGGCGTTGAAGATATGTTTGCGCACAAAC
Δ694-742 GSG fw	GACCCAGGATCTGGGAAAAAGGAAGTCGCGTTTTTTAACAAC
Δ694-742 GSG rev	CCCAGATCCTGGGTCTTTGCGCCCCATC
F423A R425A fw	TCGCCATGGCCAACATCGCGCCCAACATCTCC
F423A R425A rev	ATGTTGGCCATGGCGAGGGAGCAGGTCTTATG
R463A R465A fw	CGTGCCGGCGCCGTGACCTTCGACCGCAGTG
R463A R465A rev	ACGGCGCCGGCACGGAAAAACCTCCTCTCTGG
R425G fw	TCATGGGCAACATCGCGCCCAACATCTCC
R425G rev	ATGTTGCCCATGAAGAGGGAGCAGGTC
R470G fw	CGACGGCAGTGTTAACATTAAAGAGATCTGTTG
R470G rev	GTTAACACTGCCGTCGAAGGTCACCCAGCC
R497A R502A fw	GCCGACCTGACCCGGGCCGTTCGCAACATCAACGGCATC
R497A R502A rev	GGCCCGGGTCAGGTCGGCGTTCACACCAGGGCTCAG
W537-GSA-Q553 fw	GGGCTCCGCCCAAAACCCGATCTTGAAGAATATCACCG
W537-ASG-Q553 rev	GTTTTGGGCGGAGCCCCAAAGCTGTGTCCTGTCATCC
E567-GSGSGS-E599 fw	GGCTCCGGATCCGGGTCTGAGCGGGATGAGAAGTTGATTAAGG
E567-GSGSGS-E599 rev	AGACCCGGATCCGGAGCCTTCCTCGATCAGGTAGTCGGTG
ARS2 R854A Y859A fw	GCGGCCATTGTGGAAGCTCGGGACCTGGATGCCC
ARS2 R854A Y859A	GCTTCCACAATGGCCGCTGGGTCTCCACGAACCATCC

rev	
ARS2 F871D fw	GATTTCGATTGACTAGAGGGCCCTATTCTATAG
ARS2 F871D rev	GGCCCTCTAGTCAATCGAAATCAACATCGTCTGGGGCAT C
ARS2 845 fw	GAAACCTCGC TGA CTAGAGGGCCCTATTCTATAG
ARS2 845 rev	CCTCTAGTCA GCGAGGTTTC CCAGGATAACC

Supplementary References

1. O'Sullivan, C. et al. Mutagenesis of ARS2 Domains To Assess Possible Roles in Cell Cycle Progression and MicroRNA and Replication-Dependent Histone mRNA Biogenesis. *Molecular and Cellular Biology* **35**, 3753-3767 (2015).
2. Ishida, T. & Kinoshita, K. PrDOS: prediction of disordered protein regions from amino acid sequence. *Nucleic Acids Res* **35**, W460-4 (2007).
3. Gouet, P., Courcelle, E., Stuart, D.I. & Metoz, F. ESPript: analysis of multiple sequence alignments in PostScript. *Bioinformatics* **15**, 305-8 (1999).
4. Machida, S., Chen, H.Y. & Yuan, Y.A. Molecular insights into miRNA processing by Arabidopsis thaliana SERRATE. *Nucleic Acids Research* **39**, 7828-7836 (2011).
