# Supplementary material for: Structural analysis of human ARS2 as a platform for co-transcriptional RNA sorting
Source: Nat Commun. 2018 Apr 27;9:1701. doi: 10.1038/s41467-018-04142-7 (PMC5923425; doi:10.1038/s41467-018-04142-7)
Supplement: Supplementary file 3 — Description of Additional Supplementary Files [file 41467_2018_4142_MOESM3_ESM.pdf]

Description of Additional Supplementary Files

File Name: Supplementary Dataset 1
Description: Limma analysis of LC-MS/MS identified proteins in EGFP-ARS2 precipitates (EXCEL tale).
